# The LysR-family transcriptional regulator VtlR coordinates carbon metabolism, oxidative and nitrosative stress resistance, and virulence in *Brucella melitensis*

**DOI:** 10.1186/s13567-025-01658-x

**Published:** 2025-11-07

**Authors:** Yong Wang, Mengsi Li, Xinmei Yang, Yi Yin, Jinyue Liu, Jing Qu, Yanqing Bao, Jingjing Qi, Xiangan Han, Shaohui Wang, Mingxing Tian

**Affiliations:** 1 College of Veterinary Medicine, Anhui Agricultural University, Hefei, 230036 China; 2 Shanghai Veterinary Research Institute, Chinese Academy of Agricultural Sciences (CAAS), 518 Ziyue Road, Shanghai, 200241 China

**Keywords:** *Brucella melitensis*, LysR-family, VtlR regulon, virulence, transcriptional regulation

## Abstract

Brucellosis, a globally significant zoonotic disease caused by *Brucella* infection, relies on the pathogen’s ability to invade and replicate within host cells. This intracellular replication is tightly regulated by transcriptional networks, including the LysR-family regulator VtlR, which is critical for *B. abortus* virulence but whose role in *B. melitensis* remains unclear. Here, we constructed *vtlR* mutant and complemented strains in *B. melitensis* M5 and demonstrated that VtlR is essential for virulence. Phenotypic assays revealed that *vtlR* deletion impaired bacterial growth on L-fucose, D-glucose, and meso-erythritol, increased sensitivity to hydrogen peroxide and sodium nitroprusside, and reduced intracellular survival in RAW264.7 macrophages while triggering reactive oxygen species (ROS) production. RNA-seq and RT-qPCR analysis indicated that VtlR positively regulates small RNA AbcR2 and three DUF1127-domain proteins (RS13565, RS04310, RS13280), mirroring its regulatory role in *B. abortus*. However, overexpression of these targets failed to restore virulence in the *vtlR* mutant. Notably, the mutant strain elicited protective immunity in mice, suggesting its potential as a live-attenuated vaccine candidate. Collectively, this study elucidates the VtlR regulon in *B. melitensis*, advancing our understanding of *Brucella* pathogenesis and vaccine development.

**Supplementary Information:**

The online version contains supplementary material available at 10.1186/s13567-025-01658-x.

## Introduction

Brucellosis is one of the most common bacterial zoonotic diseases in the world, spreading widely in the world. *Brucella* infection in domestic animals can lead to miscarriage and infertility [1]. Human infection is manifested as undulant fever, which can spread throughout the body and colonize multiple parts of the body without proper or timely treatment, and is accompanied by complications, such as arthritis, splenomegaly and endocarditis [2]. This has led to huge economic losses and serious public health security problems [3].

The pathogenic *Brucella* is Gram-negative bacteria, and facultative intracellular bacterium, without classical virulence factors, such as exotoxins, cytolytics, capsules, flagella and so on [4]. Its virulence is dependent on the ability to invade and replicate within professional or non-professional phagocytes [5]. While trafficking within the macrophage, the bacteria encounter many potentially harmful conditions, including exposure to reactive oxygen and nitrogen species, destructive enzymes, low pH, nutrient deprivation and diminished oxygen levels [6, 7]. In order to adapt to the intracellular environment, *Brucella* completes the intracellular replication process by regulating the co-expression of a series of genes [8, 9]. Among them, the family of transcriptional regulators is a key component of *Brucella* regulating the expression of target genes.

LysR-family transcriptional regulators (LTTRs) are widely present in prokaryotes and can bind to the promoter region of target genes, activate or inhibit the transcription of target genes, and participate in the regulation of a variety of bacterial metabolic processes, such as virulence, metabolism, adhesion and invasion [10]. To date, several LTTRs have been reported to be associated with *Brucella* virulence, such as BvtR, LysR12, LysR13, LysR18, and VtlR [11–13]. The VtlR is required for full virulence of *B*. *abortus*, which activates the expression of the small regulatory RNA (sRNA) AbcR2 affect *B*. *abortus* virulence, and activates the expression of three small proteins BAB1_0914, BAB2_0512 and BAB2_0574 linked to fucose utilization in *B. abortus* [13, 14]. However, whether VtlR plays a similar role in the virulence of *B. melitensis* has not been reported.

In this study, we investigated the regulatory role of VtlR in *B. melitensis* and its impact on bacterial virulence. Our findings demonstrate that VtlR regulates the utilization of monosaccharides, including fucose, erythritol, and glucose, in *B. melitensis*. It also governs the *Brucella*'s resistance to oxidative and nitrosative stress, influences intracellular survival, and suppresses the release of reactive oxygen species (ROS) in activated macrophages. Furthermore, VtlR regulates the same target genes as *B. abortus*, including the small RNA AbcR2 and three small protein homologs (BM28_RS13565 [abbreviated as RS13565 hereafter], RS04310, and RS13280). However, complementation of these target genes failed to restore the virulence of the *vtlR* mutant. Additionally, we found that the *B. melitensis vtlR* mutant, as an attenuated vaccine strain, provided strong protective efficacy in a mouse model. In all, this study expands our understanding of the VtlR regulon in *Brucella* and offers valuable insights into *Brucella* pathogenesis.

## Materials and methods

### Bacterial strains, plasmids and animals

The attenuated strain *B. melitensis* M5, derived from the wild-type strain M28, was obtained from the Chinese Veterinary Culture Collection Center (CVCC, Beijing, China). All bacterial strains and plasmids used in this study are listed in Table 1. Female BALB/c mice (6–8 weeks old) were purchased from Shanghai Jsj-lab Animal Co., Ltd. and housed in individually ventilated cages (IVCs). All experiments were conducted in a biosafety level 2 (BSL-2) and Animal BSL-2 (ABSL-2) laboratories at the SHVRI, CAAS.

**Table 1:** **Bacterial strains and plasmids used in this study**

Strains and plasmids	Description	Sources
Strains		
* B. melitensis* M5	Low virulence strain; smooth phenotype	CVCC
Δ*vtlR*	The *vtlR* deletion mutant deprived from M5	This study
Δ*vtlR*-Rev	The *vtlR* complemented strain	This study
* E. coli* DH5α	F^−^, φ80d*lacZ* ΔM15, Δ*(lacZYA-argF)*U169, *recA1*, *endA1*, *hsdR17(rk*^*−*^*, mk*^+^*)*, *phoA*,*supE44*, *thi-1*, *gyrA96*, *relA1*, *λ*^*−*^	TIANGEN
Δ*vtlR*(pMT-AbcR2)	Overexpression of the *abcR2* gene in Δ*vtlR* mutant	This study
Δ*vtlR*(pMT-RS13565)	Overexpression of the *RS13565* gene in Δ*vtlR* mutant	This study
Δ*vtlR*(pMT-RS04310)	Overexpression of the *RS04310* gene in Δ*vtlR* mutant	This study
Δ*vtlR*(pMT-RS13280)	Overexpression of the *RS13280* gene in Δ*vtlR* mutant	This study
Δ*vtlR*(pMT)	The Δ*vtlR* mutant harboring the empty plasmid pMT	
Plasmids		
pKB	Kan^R^; pUC19 derived plasmid containing *sacB* gene	This study
pMT	Cm^R^, pMCR plasmid containing a *cspA* terminator	This study
pKB-Δ*vtlR*	pKB plasmid carrying the upstream and downstream homologous arm fragments of the *vtlR* gene	This study
pKB-C*vtlR*	pKB plasmid carrying the *vtlR* gene along with its upstream and downstream homologous arms	This study
pMT-AbcR2	pMT plasmid harboring the *abcR2* gene	This study
pMT-RS13565	pMT plasmid harboring the *RS13565* gene	This study
pMT-RS04310	pMT plasmid harboring the *RS04310* gene	This study
pMT-RS13280	pMT plasmid harboring the *RS13280* gene	This study

### Plasmid construction

To generate the *vtlR* deletion mutant, we employed an overlap PCR strategy to construct the suicide plasmid pKB-Δ*vtlR*, as previously described [15]. Briefly, a 750-bp upstream fragment and a 750-bp downstream fragment of the *vtlR* gene were amplified from the genomic DNA of the M5 strain using primer pairs VtlR-UF/UR and VtlR-DF/DR, respectively. These fragments were fused by overlap PCR using primers VtlR-UF and VtlR-DR. The resulting product was ligated into the linearized pKB plasmid using the ClonExpress II One Step Cloning Kit (Vazyme, Nanjing, China) and transformed into *E. coli* DH5α, yielding pKB-Δ*vtlR*.

For the *vtlR* complemented strain, we generated an in-situ restoration plasmid based on pKB as our previously reported [16]. A DNA fragment containing the upstream region, *vtlR* gene, and downstream region was amplified from the M5 genome using primers VtlR-UF and VtlR-DR. The purified fragment was ligated into linearized pKB and transformed into *E. coli* DH5α, resulting in plasmid pKB-C*vtlR*.

To generate overexpression strains for VtlR-regulated target genes, we constructed a cloning plasmid pMT by inserting an *E. coli cspA* terminator downstream of the multiple cloning site in the pMCR plasmid [17]. The target genes *RS13565*, *RS04310*, *RS13280*, and *abcR2*, along with their predicted promoter regions, were amplified using primers listed in Additional file 1. Each fragment was cloned into the BamH I/Kpn I-digested pMT plasmid using the ClonExpress II One Step Cloning Kit and transformed into *E. coli* DH5α. The resulting recombinant plasmids were designated pMT-RS13565, pMT-RS04310, pMT-RS13280, and pMT-AbcR2.

### Construction of recombinant strains

The *vtlR* deletion mutant and complemented strain were generated using the previously described *sacB*-assisted counterselection technique [15]. The suicide plasmids pKB-Δ*vtlR* (for deletion) or pKB-C*vtlR* (for complementation) were electroporated into competent *Brucella* cells. Transformants were sequentially selected on Tryptic Soy Agar (TSA) plates containing kanamycin (for plasmid integration) and 5% sucrose (for counterselection). The resulting *vtlR* deletion mutant was designated as Δ*vtlR*, while the complemented strain derived from Δ*vtlR* was named Δ*vtlR*-Rev.

### Western blotting

To further validate VtlR protein expression and LPS integrity, Western blotting was performed as previously described [11]. Briefly, the parental strain M5, Δ*vtlR*, and Δ*vtlR*-Rev were cultured overnight in Tryptic Soy Broth (TSB), harvested by centrifugation, and resuspended in 1 × SDS loading buffer, followed by boiling for 10 min. Primary antibodies included rabbit anti-VtlR polyclonal antibody (prepared in our lab), rabbit anti-GroEL polyclonal antibody (prepared in our lab), and mouse anti-O-antigen monoclonal antibody (A76/12G12/F12). IRDye 680RD Goat anti-Mouse or IRDye 800CW Goat anti-Rabbit IgG (LI-COR Biosciences, Lincoln, NE, USA) was used as the secondary antibody. Blots were visualized using an Odyssey Imaging System (LI-COR).

### Bacterial growth analysis

Bacterial growth was monitored by measuring optical density at 600 nm (OD_600_). The parental strain M5, Δ*vtlR* mutant, and complemented strain Δ*vtlR*-Rev were cultured in either TSB or a chemically defined Plommet's medium (PM) [18]. The PM composition was as follows: 2.3 g/L K_2_HPO_4_, 3 g/L KH_2_PO_4_, 0.1 g/L Na_2_S_2_O_3_, 5 g/L NaCl, 0.2 g/L niacin, 0.2 g/L thiamine, 0.07 g/L pantothenic acid, 0.5 g/L (NH_4_)_2_SO4, 0.01 g/L MgSO_4_, 0.1 mg/L MnSO_4_, 0.1 mg/L FeSO_4_, 0.1 mg/L biotin, and 1 mM methionine, supplemented with one of the following carbon sources: 2 g/L meso-erythritol, 1 g/L D-glucose, or 2 g/L L-fucose. For growth curve analysis, overnight cultures were harvested by centrifugation and resuspended in the appropriate medium to an OD_600_ of 1.0. A 500 μL aliquot of each bacterial suspension was inoculated into 4.5 mL of fresh TSB or PM, and incubated at 37 °C with shaking at 200 rpm. OD_600_ measurements were taken at 6-h intervals to monitor growth kinetics.

### Bactericidal test

The bacterial sensitivity to oxidative stress and cationic bactericidal peptides was evaluated using hydrogen peroxide (H_2_O_2_) and polymyxin B, respectively, following previously described methods [11]. Mid-logarithmic phase cultures (OD_600_ ≈ 1.0) of the parental strain M5 and its derivatives grown in TSB were diluted in phosphate-buffered saline (PBS) to 5 × 10^5^ CFU/mL. Aliquots (50 μL) of bacterial suspension were mixed with equal volumes of either H_2_O_2_ (final concentrations: 1.0, 2.0, or 4.0 mM) or polymyxin B (final concentrations: 0.5, 1, or 2 mg/mL). After incubation at 37 °C with shaking (200 rpm) for 1 h, viable bacteria were quantified by plating on TSA. For acid tolerance assessment, bacterial suspensions (50 μL) were inoculated into 950 μL TSB adjusted to pH 7.2, 6.5, 5.5, or 4.5 and incubated at 37 °C with shaking (200 rpm) for 2 h before CFU enumeration. Serum resistance was evaluated by incubating bacterial suspensions (50 μL) with either 950 μL non-inactivated goat serum, heat-inactivated goat serum, or PBS at 37 °C for 1.5 h [15]. Bacterial CFU was enumerated on TSA. Survival rates were calculated as: (CFU treatment /CFU control) × 100%, where CFU treatment represents colonies from treated samples and CFU control from PBS-treated samples.

Besides, nitrosative stress sensitivity was assessed using sodium nitroprusside (SNP) [11]. Bacterial suspensions (5 × 10⁹ CFU/mL) were serially diluted tenfold, and 2 μL aliquots were spotted on TSA plates containing 0.5 mM SNP. Plates were incubated at 37 °C for 3–5 days, with TSA serving as growth control.

All experiments were performed in triplicate, with results expressed as mean percentage survival ± standard deviation from one representative experiment.

### Cell infection

To assess *Brucella* infection with host cells, RAW264.7 cells (2.5 × 10^5^ cells/well) or HeLa cells (7 × 10^4^ cells/well) were seeded in 24-well plates and cultured to confluence. After washing twice with PBS, monolayers were infected with M5, Δ*vtlR*, or Δ*vtlR*-Rev at multiplicities of infection (MOI) of 100:1 for RAW264.7 cells or 500:1 for HeLa cells. Plates were centrifuged at 400 × *g* for 5 min to synchronize infection, followed by incubation at 37 °C for 1 h. For adhesion assessment, cells were washed five times with PBS to remove non-adherent bacteria, then lysed with 0.25% Triton X-100 in sterile water. Viable bacteria were quantified by plating on TSA. To evaluate invasion, infected cells were treated with DMEM containing 100 μg/mL gentamicin for 1 h to eliminate extracellular bacteria, washed five times with PBS, then lysed as above for bacterial enumeration. For intracellular survival analysis, following extracellular bacterial clearance, infected cells were maintained in DMEM with 2% FBS and 50 μg/mL gentamicin. At 1, 24, 48, and 72 hours post-infection (hpi), cells were lysed and intracellular bacteria were quantified by plating on TSA.

### Measurement of intracellular ROS and RNS

Intracellular reactive oxygen species (ROS) and reactive nitrogen species (RNS) levels were measured using the fluorogenic substrate 2′,7′-dichlorofluorescein diacetate (DCFH-DA; Beyotime, Shanghai, China) and BBoxiProbe^®^ O52 (BestBio, Shanghai, China), respectively. RAW264.7 cells were seeded in 96-well plates at a density of 6.25 × 10^4^ cells/well and cultured for 24 h. After washing twice with PBS, the cells were infected with M5 and its derivatives at a MOI of 100:1 as described above. At 48 hpi, 100 μL of DCFH-DA (diluted 1:1000) or O52 (diluted 1:100) was added to each well, followed by incubation at 37 °C for 30 min. Subsequently, the cells were washed twice with PBS and resuspended in 200 μL of PBS or HBSS. ROS and RNS levels were quantified using a BioTek Synergy H1 Multimode Reader (Agilent Technologies, Santa Clara, CA, USA) with excitation/emission wavelengths of 488/525 nm (ROS) and 488/516 nm (RNS), respectively.

### RNA extraction, RNA sequencing (RNA-seq) and quantitative PCR (qPCR)

Total RNA was extracted from M5 strain and its derivatives using the PureLink™ RNA Mini Kit (Ambion; Thermo Fisher Scientific, Waltham, MA, USA), followed by DNase treatment with the TURBO DNA-free™ kit (Invitrogen; Thermo Fisher).

For RNA-seq analysis, ribosomal RNA was removed from total RNA using the RiboCop rRNA Depletion Kit for Mixed Bacterial Samples (Lexogen, USA), followed by fragmentation of mRNA into approximately 200 nt fragments. Double-stranded cDNA was synthesized using random hexamer primers (Illumina, San Diego, USA), with dUTP incorporated during second-strand synthesis to maintain strand specificity. The cDNA was subsequently processed through end-repair, phosphorylation, and A-tailing according to the Illumina library construction protocol. Stranded RNA-seq libraries were prepared using the Illumina Stranded mRNA Prep Kit and sequenced in paired-end mode (2 × 150 bp) on the NovaSeq 6000 platform (Illumina). Raw sequencing data were processed through base calling and quality assessment. Clean reads were obtained by removing low-quality sequences (quality score < 20), reads containing more than 10% ambiguous bases (N), and adapter-contaminated reads. All bioinformatics analyses were performed on the Majorbio Cloud Platform (Shanghai Majorbio Bio-pharm Technology Co., Ltd). Transcript abundance was quantified using RSEM (RNA-Seq by Expectation–Maximization), which employs a maximum likelihood estimation approach to account for read mapping ambiguity among isoforms, fragment length distribution, and sequence quality. Gene expression levels were normalized and reported as TPM (Transcripts Per Million) values to enable cross-sample comparisons. Differentially expressed genes were identified using DESeq2 with an adjusted *p*-value < 0.05.

For RT-qPCR, RNA was reverse-transcribed into cDNA using the PrimeScript RT Reagent Kit (Takara Bio, Kusatsu, Shiga, Japan) under the following conditions: 37 °C for 20 min, followed by 85 °C for 10 s. qPCR was performed using the 2 × SYBR qPCR Master Mix (Vazyme) on a QuantStudio 3 system (ABI, Thermo Fisher) with the following cycling parameters: 95 °C for 2 min, then 40 cycles of 95 °C for 15 s and 60 °C for 1 min. Each gene was analyzed in triplicate, and relative transcript levels were calculated using the 2^−ΔΔCt^ method, with glyceraldehyde-3-phosphate dehydrogenase (GAPDH) as the reference gene. All RT-qPCR primers are listed in Additional file 1.

### Mouse infection assay

The virulence of M5 and its derivative strains was evaluated in 6-week-old female BALB/c mice using a modified version of a previously described method [11, 15]. Bacterial cultures were grown to OD_600_ = 1.0 (~5 × 10⁹ CFU/mL) and mice were intraperitoneally inoculated with 1 × 10⁶ CFU (PBS-injected mice served as controls). At 2 and 4 weeks post-infection (wpi), five mice per group were euthanized. Spleens were aseptically collected, weighed, and homogenized in 3 mL of PBS containing 0.25% Triton X-100. Serial tenfold dilutions of the homogenates were plated on TSA for bacterial enumeration.

### Histopathological analysis

To assess the pathogenicity of strains M5, Δ*vtlR*, and Δ*vtlR*-Rev, histopathological analysis was conducted on infected mouse livers collected at 2 and 4 wpi. Tissues were fixed in 4% paraformaldehyde for 24 h and processed for sectioning and hematoxylin–eosin (HE) staining by Wuhan Servicebio Technology Co., Ltd. (Wuhan, China). Microscopic examination at various magnifications revealed characteristic pathological changes, including granulation tissue formation and other inflammatory lesions. Representative images of typical pathological features are presented, with arrows indicating key abnormalities.

### Statistical analysis

Statistical analyses were performed using GraphPad Prism 9.5 (GraphPad Software, San Diego, CA, USA). For comparisons between two groups, a Student’s *t*-test was used, while one-way or two-way ANOVA followed by Dunnett’s multiple comparisons test was applied for multi-group analysis. A *p*-value < 0.05 was considered statistically significant.

## Results

### The *vtlR* deletion and complemented strains were successfully constructed.

**Figure 1:**
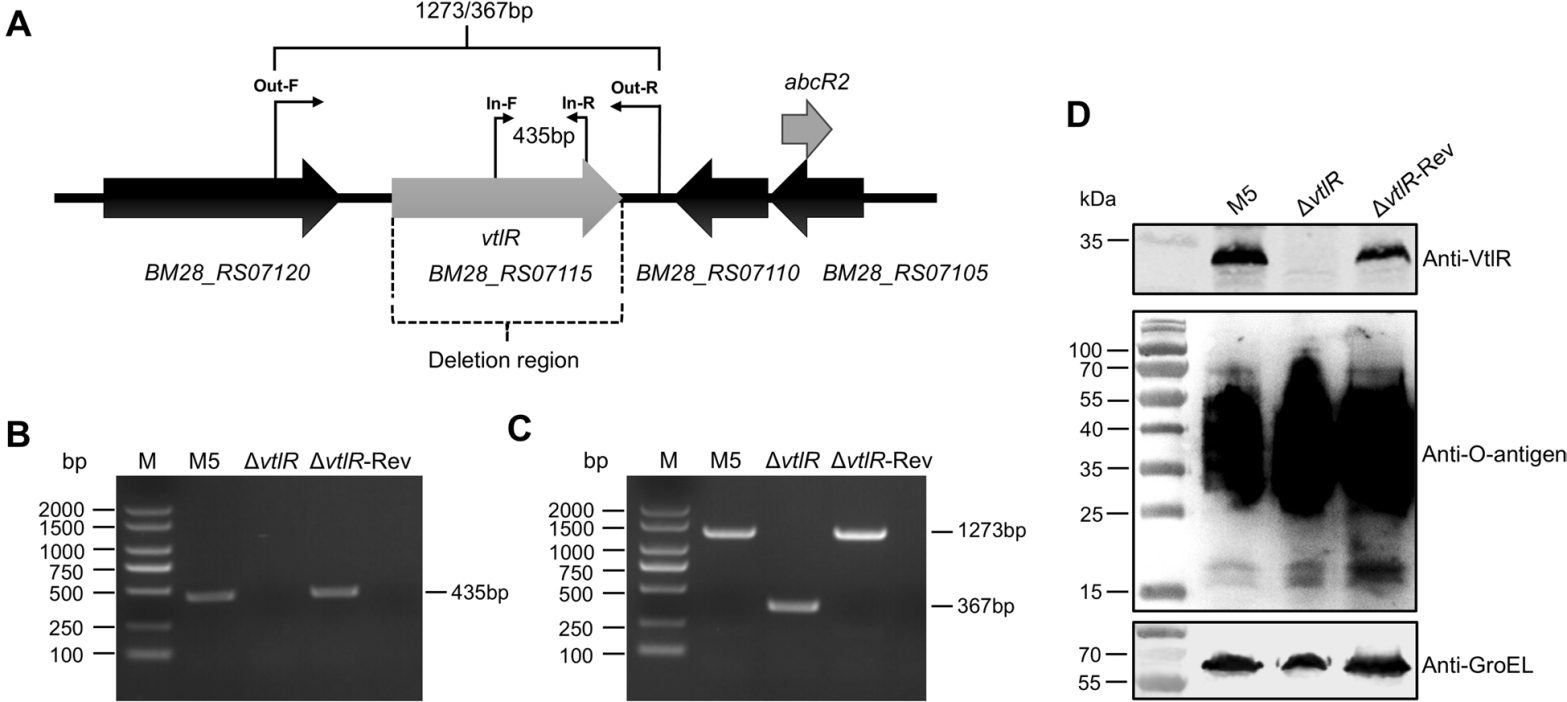
**Construction and verification of the *vtlR* deletion mutant and complemented strain**. **A** Genomic organization of the *vtlR* locus, with primer positions indicated for mutant verification. **B** PCR verification of the Δ*vtlR* mutant and complemented strain (Δ*vtlR*-Rev) using internal primers In-F/R. **C** PCR confirmation of Δ*vtlR* and Δ*vtlR*-Rev using flanking primers Out-F/R. **D** Western blot analysis of VtlR protein and O-antigen expression in the parental strain M5, Δ*vtlR* mutant, and Δ*vtlR*-Rev complemented strain.

The *B. melitensis* VtlR is encoded by the *RS07115* gene and belongs to the LysR-family transcriptional regulators. This gene is 906 bp in length and encodes a 301-amino acid protein. According to InterPro database annotations, the VtlR protein contains an N-terminal helix-turn-helix DNA-binding domain (amino acids 7–66) and a C-terminal substrate-binding domain (amino acids 95–292). The upstream gene *RS07120* encodes a thioredoxin-disulfide reductase, while the downstream genes *RS07110* and *RS07105* encode two hypothetical proteins (Figure 1A). Notably, the antisense strand of *RS07105* encodes the small RNA gene *abcR2*, which is one of the primary target genes regulated by VtlR [13]. In this study, we constructed a *vtlR* deletion mutant (Δ*vtlR*) using *B. melitensis* M5 as the parental strain, in which the entire *vtlR* gene was deleted. Considering that VtlR may regulate *abcR2* through neighboring gene effects, we also generated a complemented strain (Δ*vtlR*-Rev) by restoring the *vtlR* gene to its native locus via homologous recombination. First, we verified the Δ*vtlR* and Δ*vtlR*-Rev strains by PCR. Using the In-F/R primer pair, a 435-bp band was amplified from the parental and complemented strains but not from the deletion mutant (Figure 1B). With the Out-F/R primer pair, the parental and complemented strains yielded a 1273-bp product, whereas the Δ*vtlR* strain produced a 367-bp fragment due to gene deletion (Figure 1C). To further confirm *vtlR* deletion and complementation, we analyzed VtlR protein expression by immunoblotting. The results demonstrated successful VtlR expression in the parental and complemented strains, while no detectable signal was observed in the Δ*vtlR* mutant (Figure 1D). Additionally, given that spontaneous mutations in *Brucella* lipopolysaccharide O-antigen can lead to nonspecific attenuation [19], we evaluated O-antigen integrity by immunoblotting. As shown in Figure 1D, no O-antigen deficiency was detected in any of the three strains. Collectively, these experimental data confirm the successful construction of isogenic *vtlR* deletion and complemented strains in *B. melitensis*.

### The VtlR is essential for the full virulence of *B. melitensis*

**Figure 2:**
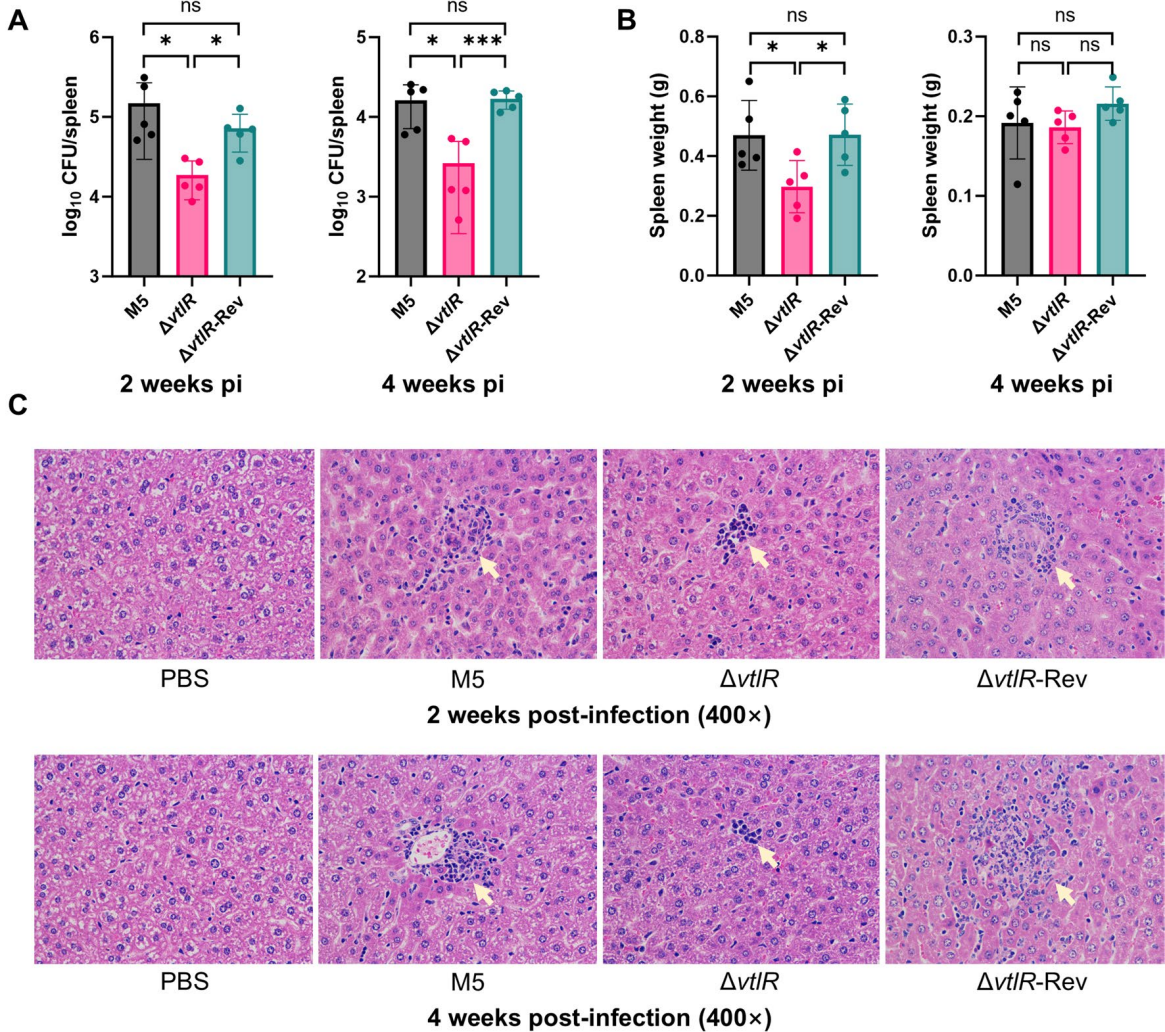
**VtlR is required for full virulence of *B. melitensis***. **A** Bacterial loads in spleens of mice infected with parental strain M5, Δ*vtlR* mutant, or Δ*vtlR*-Rev complemented strain at 2 and 4 weeks post-infection (wpi.). **B** Splenomegaly assessment by spleen weight measurement. **C** Histopathological analysis of liver sections from mice infected with M5, Δ*vtlR*, or Δ*vtlR*-Rev at 2 wpi. Statistical significance was determined using one‐way ANOVA (*p < 0.05, ***p < 0.001, ns, not significant).

To evaluate the role of VtlR in *B*. *melitensis* virulence, we assessed bacterial loads in the spleen, splenomegaly, and hepatic granuloma formation in BALB/c mice infected with the parental strain M5, Δ*vtlR* mutant, or Δ*vtlR*-Rev complemented strains at 2 and 4 wpi. The Δ*vtlR* mutant exhibited significantly reduced splenic bacterial loads compared to the M5 at both time points (*p* < 0.05), while the complemented strain (Δ*vtlR*-Rev) restored colonization to M5 levels (Figure 2A). Splenomegaly, quantified by spleen weight, was markedly attenuated in Δ*vtlR*-infected mice at 2 wpi relative to M5 and Δ*vtlR*-Rev groups (Figure 2B). By 4 wpi, spleen weights converged (~0.2 g) across all groups, suggesting attenuated strain-specific differences during chronic infection. Notably, Δ*vtlR*-infected mice developed smaller hepatic granulomas than M5- or Δ*vtlR*-Rev-infected animals, further supporting a role for VtlR in *B*. *melitensis* virulence (Figure 2C). Collectively, these findings demonstrate that VtlR is required for full virulence of *B. melitensis*.

### The *vtlR* deletion reduced the utilization of fucose, erythritol, and glucose in *B. melitensis*

**Figure 3:**
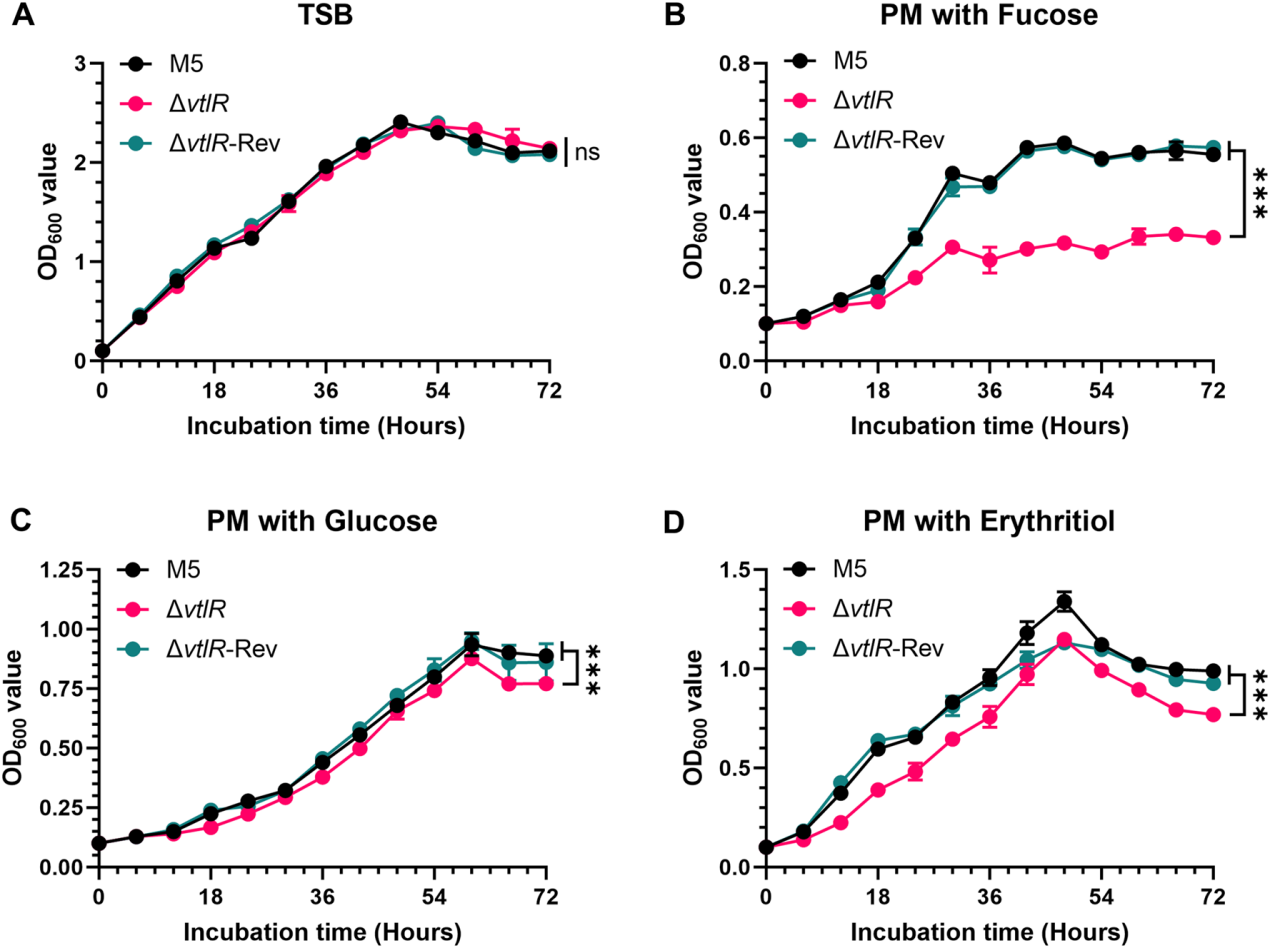
**Growth kinetics of *Brucella* strains in different culture media**. **A** Growth curves in tryptic soy broth (TSB); **B** Growth in Plommet's medium (PM) supplemented with L-fucose; **C** Growth in PM with D-glucose; **D** Growth in PM with meso-erythritol. Statistical significance was determined using two‐way ANOVA (***p < 0.001, ns, not significant).

Previous studies have demonstrated that *vtlR* deletion impairs fucose utilization in *B. abortus* [14]. To determine whether *vtlR* plays a similar role in *B. melitensis*, we evaluated the growth of the *vtlR* mutant in TSB or PM supplemented with L-fucose as the sole carbon source. Initial growth assays in nutrient-rich TSB revealed no significant difference between the *vtlR* mutant and the parental strain (Figure 3A), indicating that *vtlR* is dispensable for growth under nutrient-rich conditions. However, in the PM with L-fucose, the *vtlR* mutant exhibited significantly impaired growth compared to the parental strain (Figure 3B), suggesting a critical role for *vtlR* in fucose metabolism. Given this finding, we further assessed the impact of *vtlR* deletion on the utilization of glucose and erythritol, two carbon sources associated with *Brucella* virulence [20, 21]. Growth curve analysis showed that the *vtlR* mutant also had reduced growth with glucose and erythritol, though the defect was less pronounced than with fucose (Figures 3C, D).

### The *vtlR* deletion enhanced the sensitivity of *B. melitensis* to H₂O₂ and SNP

**Figure 4:**
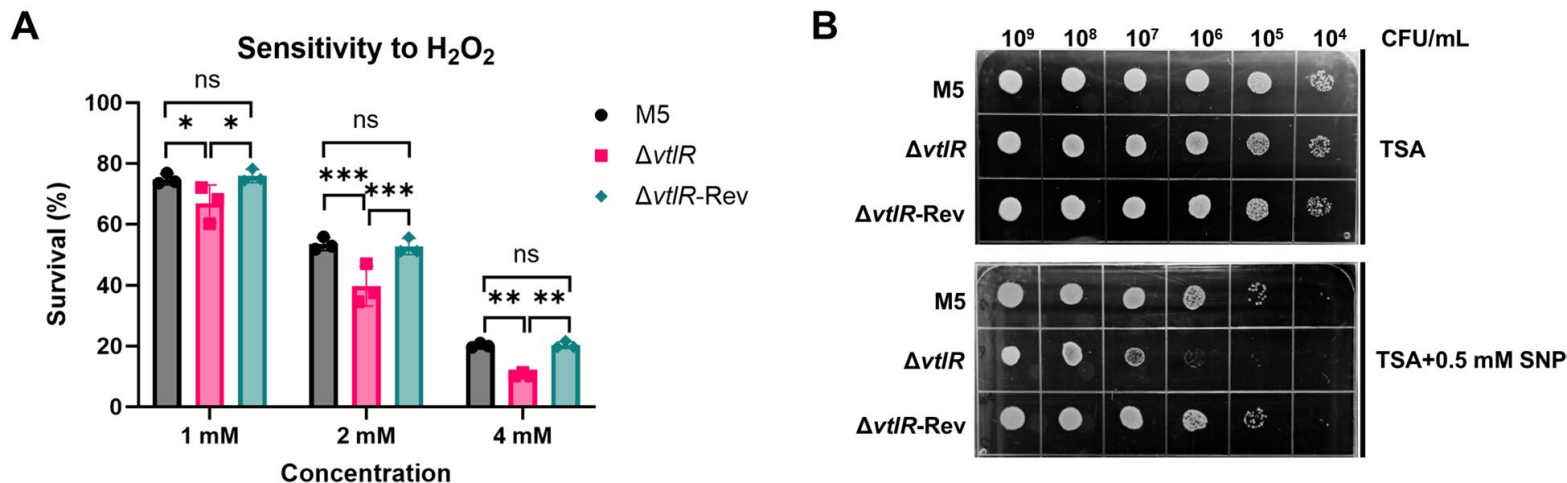
**Sensitivity of *Brucella* to oxidative killing**. **A** Hydrogen peroxide (H₂O₂) susceptibility; **B** Sodium nitroprusside (SNP) susceptibility. Statistical significance was determined using one‐way ANOVA (*p < 0.05, **p < 0.01, ***p < 0.001, ns, not significant).

To evaluate the role of VtlR in stress resistance, we compared the susceptibility of the Δ*vtlR* mutant to various bactericidal agents—including H₂O₂, SNP, polymyxin B, SDS, acidic pH, and goat serum—with that of the parental strain M5 and the complemented strain Δ*vtlR-Rev*. The Δ*vtlR* mutant exhibited significantly increased sensitivity to both H₂O₂ and SNP compared to the M5 and Δ*vtlR*-Rev strains (*p* < 0.05; Figures 4A, B), indicating impaired oxidative and nitrosative stress resistance. In contrast, no notable differences were observed in susceptibility to polymyxin B, SDS, acidic pH, or natural goat serum among the strains (Additional file 2). These findings demonstrate that VtlR is critical for *Brucella*’s defense against oxidative and nitrosative stress but dispensable for resistance to membrane-disrupting agents, acidic stress, or serum-mediated killing.

### The *vtlR* mutant reduced intracellular survival and induced macrophage ROS production

**Figure 5:**
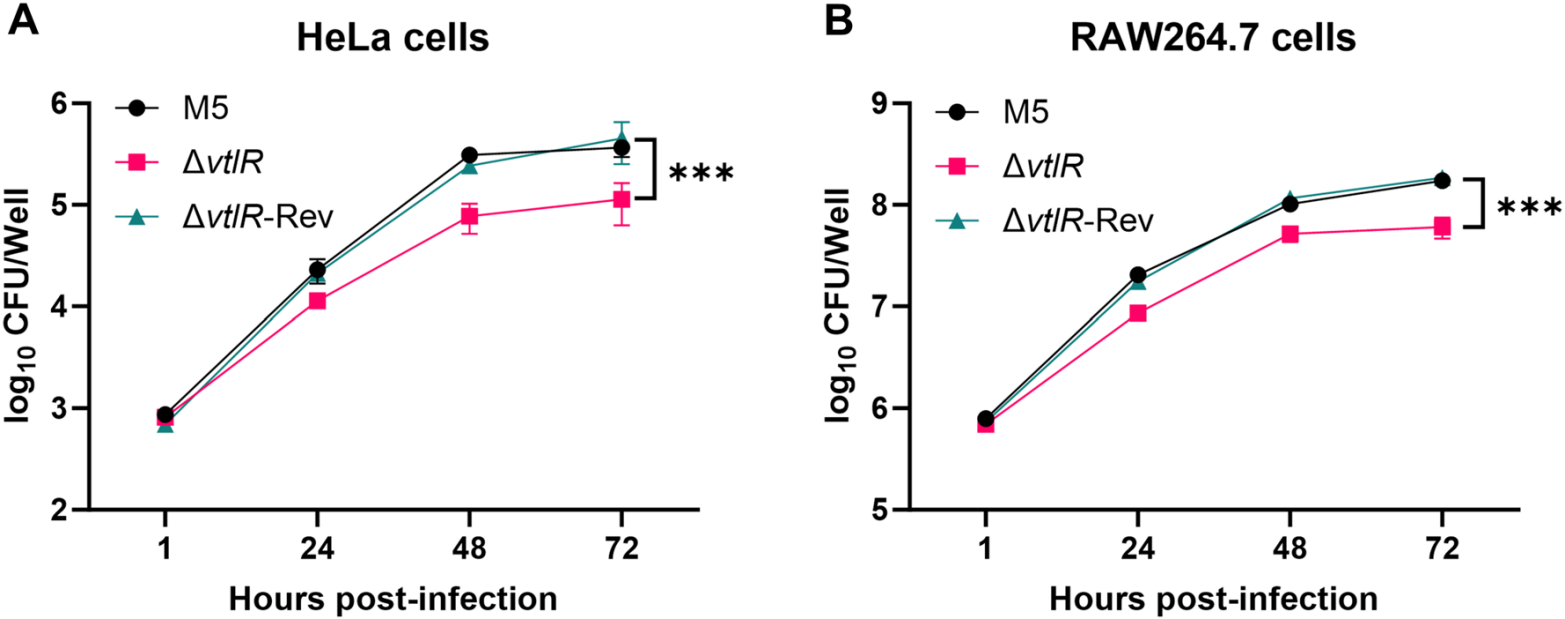
**Intracellular survival capacity of *Brucella* strains**. **A** Survival of parental strain M5, Δ*vtlR* mutant, and complemented strain Δ*vtlR*-Rev in HeLa cells; **B** Survival of M5, Δ*vtlR*, and Δ*vtlR*-Rev in RAW 264.7 macrophages. Statistical significance was determined by two-way ANOVA (***p < 0.001).

Host cell invasion is a critical step in *Brucella*’s establishment of intracellular infection. To determine whether VtlR contributes to this process, we assessed the adherence and invasion capacities of the Δ*vtlR* mutant in HeLa and RAW264.7 cells. Both assays revealed that the Δ*vtlR* mutant adhered to and invaded these cells as efficiently as the parental M5 and complemented Δ*vtlR*-Rev strains (Additional file 3), indicating that VtlR is dispensable for early infection steps. Since intracellular survival is essential for *Brucella* virulence, we further evaluated the replication of the Δ*vtlR* mutant within host cells. Strikingly, the mutant exhibited significantly impaired intracellular survival in both HeLa and RAW264.7 macrophages compared to the parental and complemented strains (*p* < 0.05; Figures 5A, B). These results demonstrate that VtlR plays a pivotal role in *B. melitensis*’s ability to proliferate within host cells but is not required for initial adherence or invasion.

**Figure 6:**
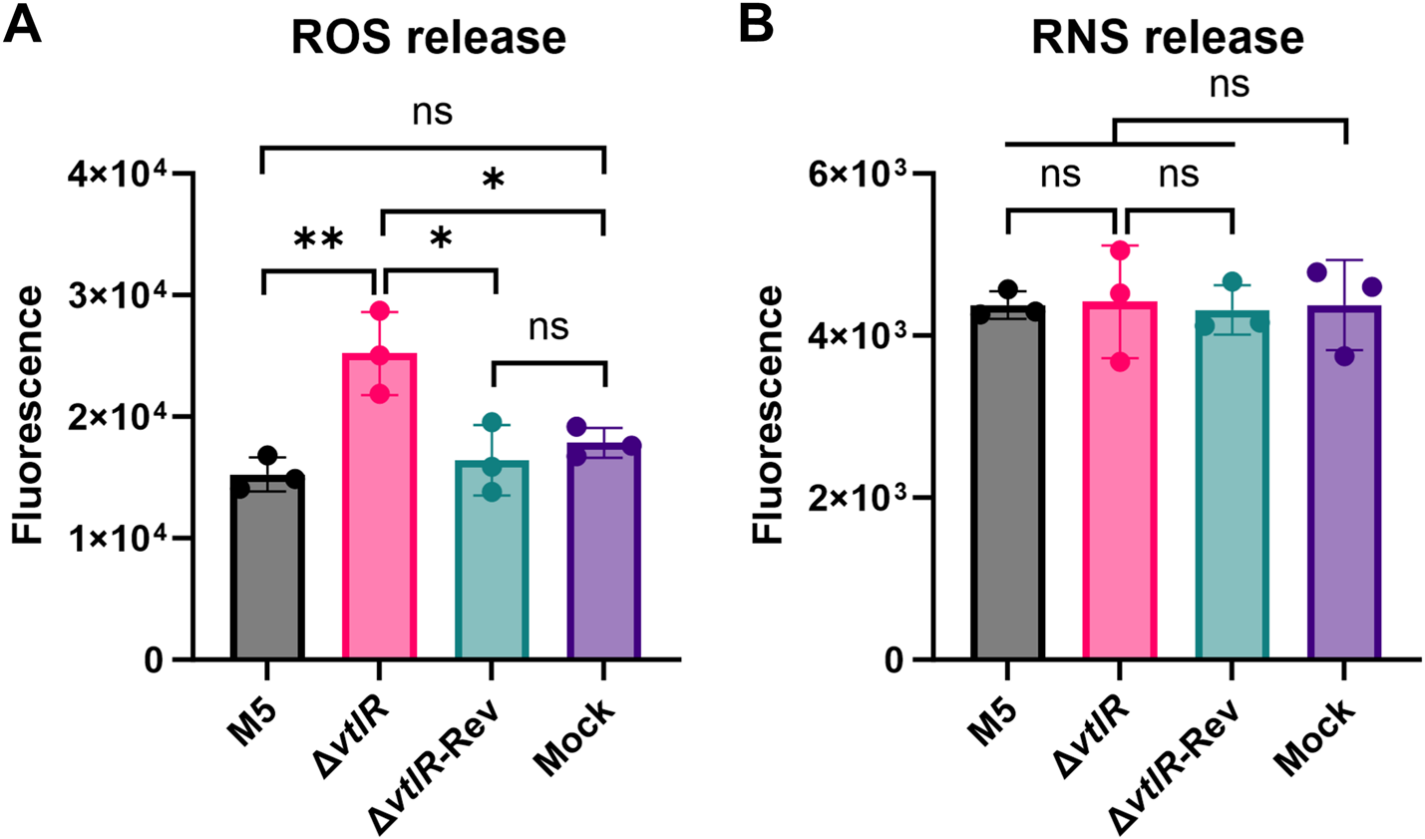
**ROS and RNS production in *Brucella*-infected macrophages**. **A** Intracellular ROS levels in RAW 264.7 macrophages infected with M5, Δ*vtlR*, or Δ*vtlR*-Rev at 48 h post-infection (hpi). **B** Nitric oxide (NO) production in infected RAW 264.7 macrophages at 48 hpi. Statistical significance was determined using one‐way ANOVA (*p < 0.05, **p < 0.01, ns, not significant).

Based on the Δ*vtlR* mutant's increased sensitivity to H₂O₂ and SNP *in vitro* and its defective intracellular survival, we postulated that infection with this mutant might elicit enhanced ROS and RNS production in macrophages. To test this hypothesis, we quantified ROS and RNS levels in RAW264.7 macrophages at 48 hpi with either the parental strain M5, Δ*vtlR* mutant, or complemented strain Δ*vtlR*-Rev. Notably, macrophages infected with the Δ*vtlR* mutant exhibited significantly elevated ROS levels compared to those infected with either the M5 or complemented strains (*p* < 0.05; Figure 6A). In contrast, RNS production remained comparable across all infection groups (Figure 6B). These results demonstrate that VtlR deficiency enhances ROS, but not RNS, production in infected macrophages.

### Identification of key target genes regulated by the VtlR in *B. melitensis*

**Figure 7:**
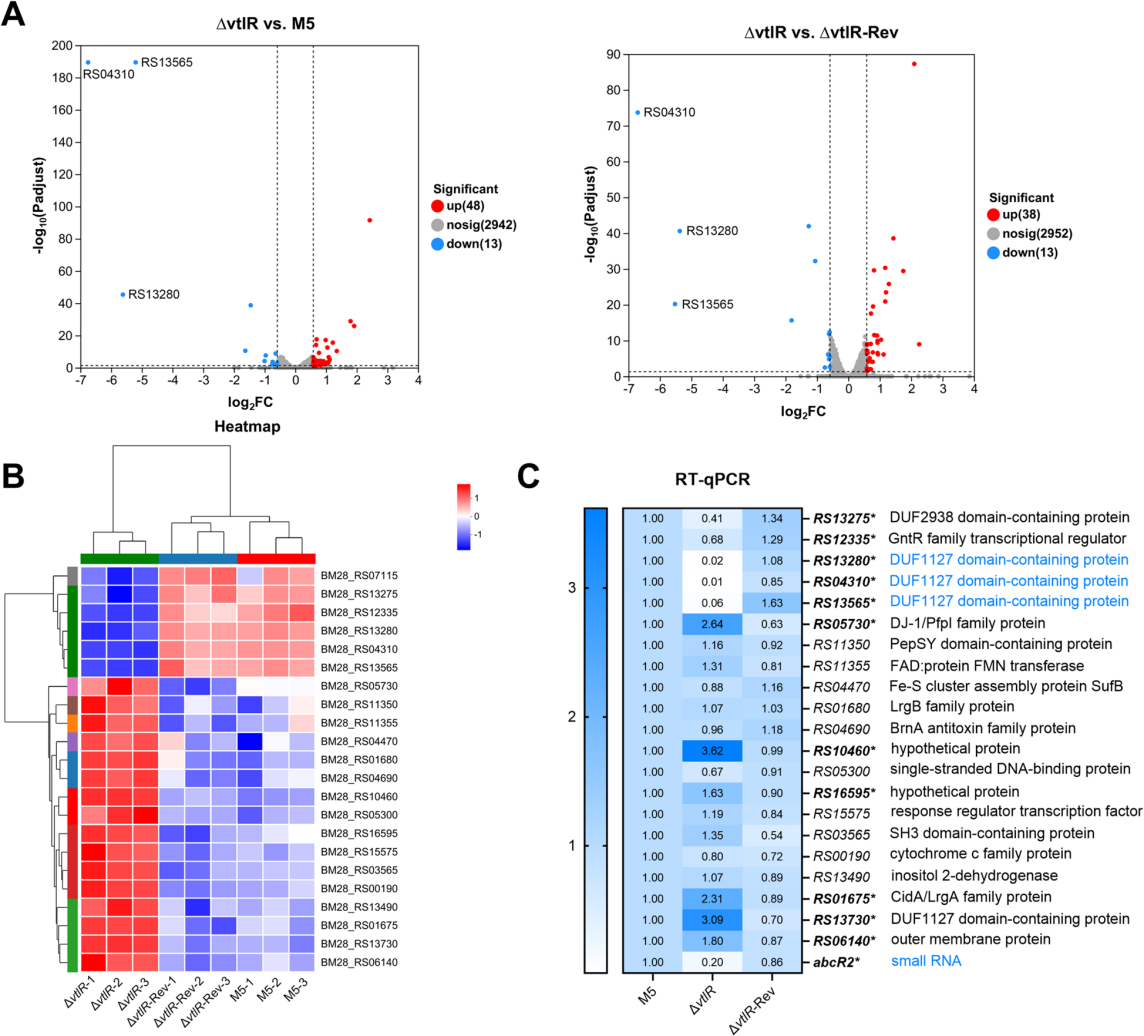
**Transcriptomic profiling of the Δ*vtlR *mutant strain**. **A** Volcano plots of RNA-seq data showing differentially expressed genes (DEGs) in Δ*vtlR* versus parental strain M5 (left panel) and Δ*vtlR* versus complemented strain Δ*vtlR*-Rev (right panel). **B** Heatmap visualization of the 22 core DEGs common to both comparisons. **C** Experimental validation of selected DEGs by RT-qPCR. Statistical significance was determined using one‐way ANOVA (*p < 0.05).

To identify key target genes regulated by VtlR, we performed comparative RNA-seq analysis of differentially expressed genes (DEGs) among the parental strain, the Δ*vtlR* mutant, and the complemented strain Δ*vtlR*-Rev. Compared to the parental strain, the Δ*vtlR* mutant exhibited 61 DEGs (48 up-regulated and 13 down-regulated), while 51 DEGs (38 up-regulated and 13 down-regulated) were identified relative to the Δ*vtlR*-Rev strain (Figure 7A). Heatmap analysis confirmed consistent expression patterns across three biological replicates for each strain, supporting data reproducibility (Figure 7B). Further analysis focused on DEGs common to both comparisons (Δ*vtlR* mutant vs. parental and Δ*vtlR* mutant vs. Δ*vtlR*-Rev strains), revealing 22 genes (16 up-regulated and 6 down-regulated), including *RS07115* (encoding VtlR itself) (Figure 7B). RT-qPCR validation of the remaining 21 DEGs confirmed significant expression alterations in 11 genes, including 5 down-regulated and 6 up-regulated ones (Figure 7C). Notably, three genes—*RS13280*, *RS04310*, and *RS13565*—exhibited extremely significant downregulation, with fold changes of approximately 50-, 100-, and 17-fold, respectively. This is consistent with previously reported VtlR-regulated targets (*BAB1_0914*, *BAB2_0574*, *BAB2_0512*) in *B. abortus* [13]. In contrast, the fold changes of the remaining 8 genes were all less than fourfold (Figure 7C). Given prior evidence that VtlR regulates the small RNA *abcR2* in *B. abortus* [13], we evaluated *abcR2* expression in the Δ*vtlR* mutant and observed an approximate fivefold downregulation (Figure 7C). Collectively, these results indicate that VtlR predominantly modulates the expression of three DUF1127 domain-containing proteins and the small RNA AbcR2 in *B. melitensis*. Therefore, these four genes were selected for further investigation in subsequent studies.

### VtlR-regulated target gene overexpression fails to complement Δ*vtlR* virulence defects

In a previous study, Sheehan et al. constructed single-gene deletion mutants and a quadruple-gene deletion mutant targeting four genes *(BAB1_0914*, *BAB2_0574*, *BAB2_0512*, and *abcR2*) in *B. abortus* strain 2308. Using macrophage infection assays, they confirmed that these genes are not involved in the intracellular survival of *B. abortus* [13]. Moreover, Budnick et al. demonstrated via mouse infection experiments that single, triple, or quadruple deletion of these four genes does not impact the virulence of *B. abortus* [14]. Based on these studies, we note that assessing bacterial virulence via gene deletion in wild-type strains is not directly linked to the VtlR regulatory mechanism.

**Figure 8:**
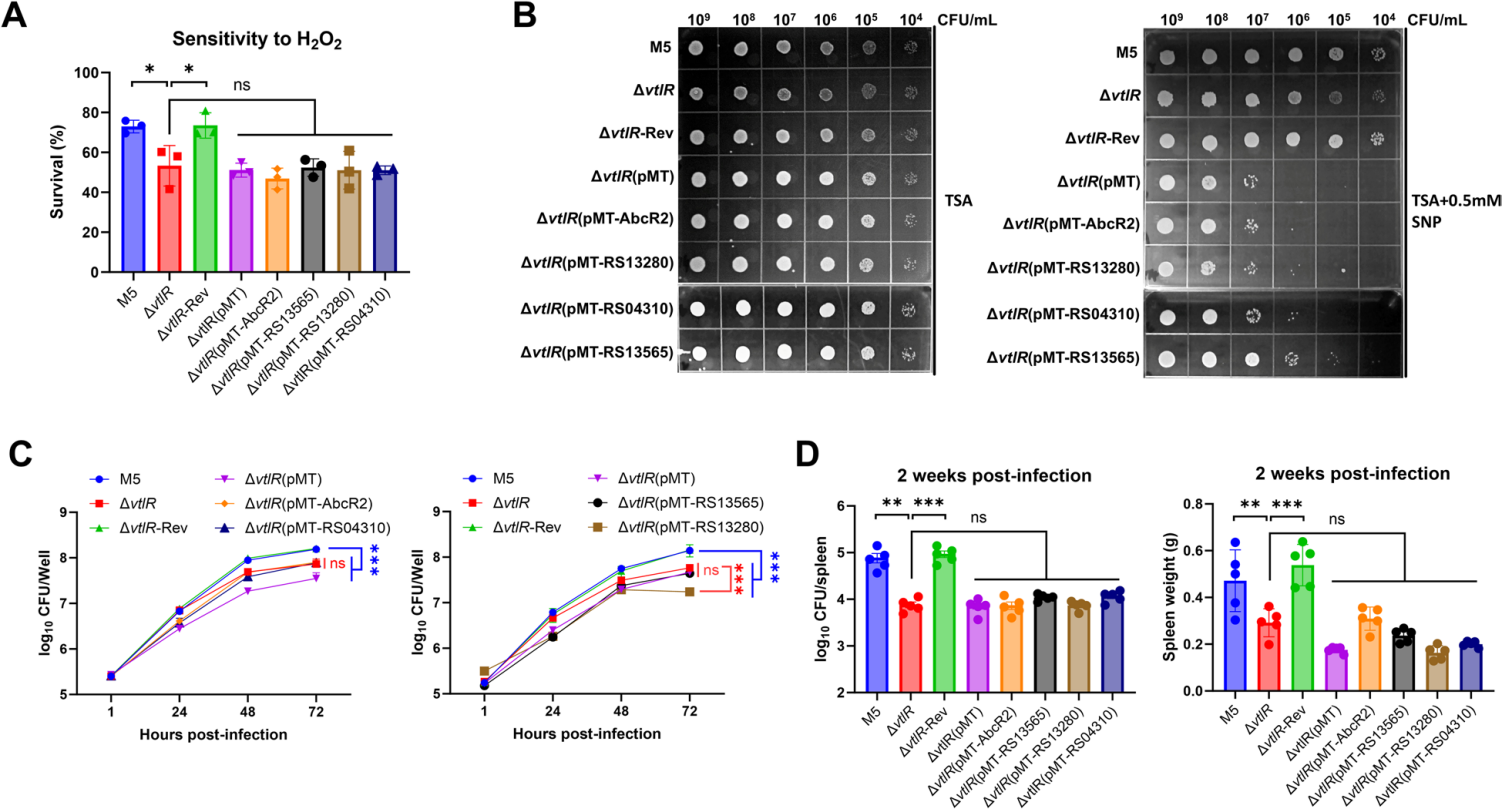
**Functional complementation analysis of VtlR-regulated target genes**. **A** H_2_O_2_ sensitivity assay showing failed phenotypic complementation in the Δ*vtlR* mutant overexpressing target genes. **B** SNP susceptibility test demonstrating unaltered stress sensitivity in overexpressed strains. **C** Intracellular survival capacity in macrophages remains attenuated despite target gene overexpression. **D** Murine infection experiments confirm persistent virulence attenuation of the Δ*vtlR* mutant with target gene overexpression. Statistical significance was determined by one-way or two-way ANOVA (*p < 0.05, **p < 0.01, ***p < 0.001, ns, not significant).

In the present study, these four genes exhibited significantly downregulated expression in the Δ*vtlR* mutant. Thus, we aimed to specifically characterize the function of each gene in the context of *vtlR* deletion by restoring or overexpressing individual genes. To this end, we complemented the Δ*vtlR* strain with *RS13280*, *RS04310*, *RS13565*, and *abcR2* using the pMT plasmid, generating the strains Δ*vtlR*(pMT-*RS13280*), Δ*vtlR*(pMT-*RS04310*), Δ*vtlR*(pMT-*RS13565*), and Δ*vtlR*(pMT-*abcR2*). A control strain, Δ*vtlR*(pMT), harboring the empty pMT vector, was also constructed. RT-qPCR analysis confirmed successful overexpression of the target genes in the complemented strains, with expression levels elevated by 20- to 60-fold compared to the control (Additional file 4). We next assessed the effects of gene overexpression on *Brucella* virulence phenotypes. H_2_O_2_ and SNP tolerance assays revealed that none of the complemented strains rescued the hypersensitivity of the Δ*vtlR* mutant to oxidative stress (Figures 8A, B). Similarly, intracellular survival assays demonstrated that overexpression of *RS13280*, *RS04310*, *RS13565*, or *abcR2* failed to restore the replication defect of the Δ*vtlR* mutant within macrophages. Notably, RS13280 overexpression further impaired intracellular survival compared to the Δ*vtlR* mutant, and all overexpressed strains exhibited significant survival defects relative to the parental and Δ*vtlR*-Rev strains (Figure 8C). In vivo infection experiments further supported these findings. Mice infected with the overexpressed strains showed splenic bacterial loads comparable to those infected with the Δ*vtlR* mutant, with significantly reduced colonization compared to the parental and complemented *vtlR* strains. Additionally, spleen weights in mice infected with the overexpressed strains did not differ significantly from those infected with the Δ*vtlR* mutant, but both groups exhibited markedly reduced spleen weights compared to animals infected with the parental or Δ*vtlR*-Rev strains. Taken together, these results indicate that overexpression of *RS13280*, *RS04310*, *RS13565*, or *abcR2* does not restore the virulence attenuation caused by *vtlR* deletion in *B. melitensis*.

### Vaccination with the Δ*vtlR* mutant induces strong immunoprotection in murine model

**Figure 9:**
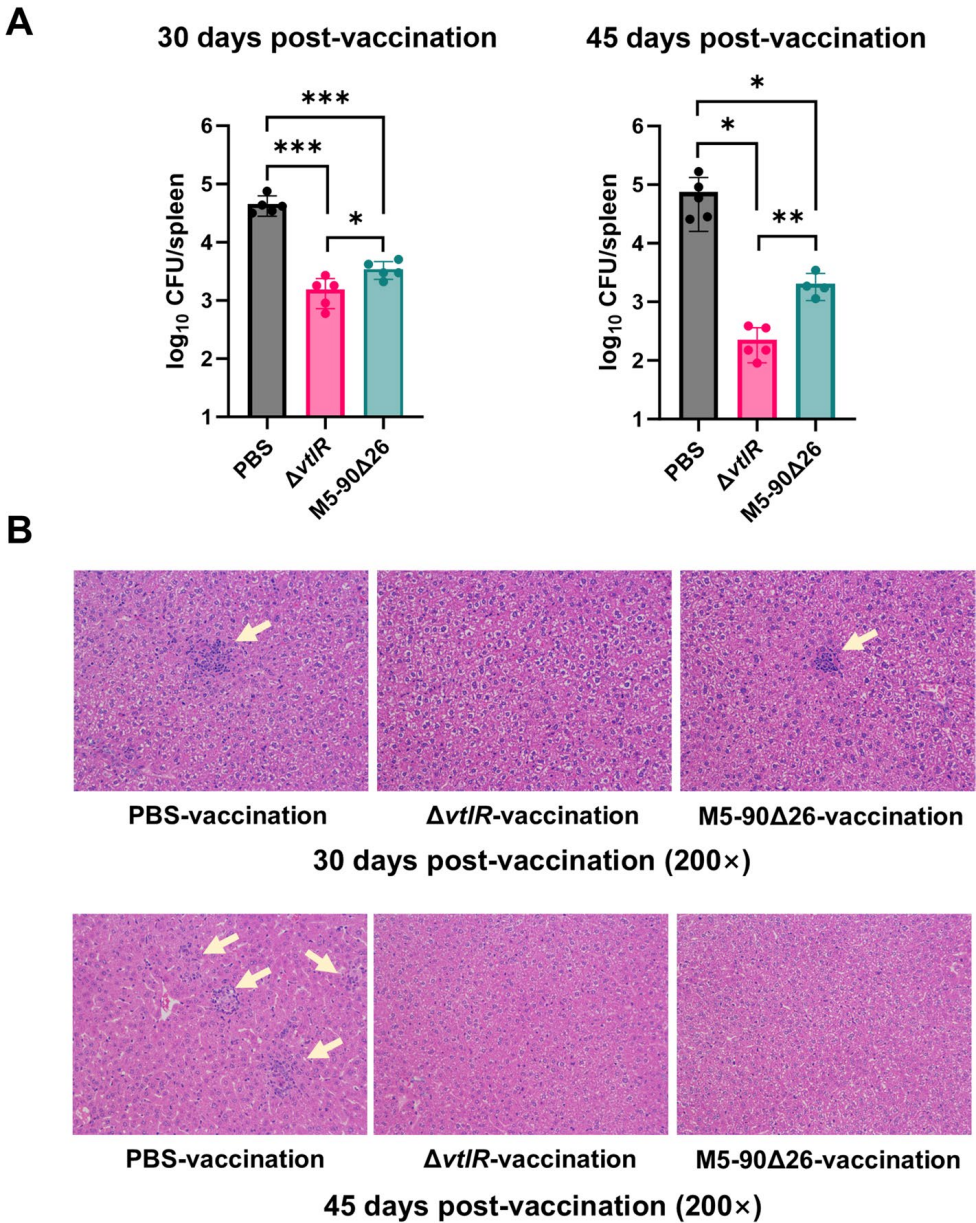
**Evaluation of immunoprotection conferred by the Δ*vtlR* mutant in a murine model**. **A** Bacterial burden in the spleen of mice challenged with the parental strain M5 at 30 and 45 days post-vaccination (dpv). **B** Histopathological analysis of liver tissue from mice challenged with the parental strain at 30 and 45 dpv. Statistical significance was determined by one-way ANOVA (*p < 0.05, **p < 0.01, ***p < 0.001, ns, not significant).

Given the marked attenuation of virulence in the *vtlR* mutant, we investigated its potential as a live-attenuated vaccine candidate against *Brucella*. In this study, mice were immunized with the *vtlR* mutant (10^5^ CFU), using the commercial vaccine strain M5-90Δ26 as a positive control and PBS as a negative control. Protective efficacy was assessed by challenging mice with the parental strain M5 at 30 and 45 days post-vaccination (dpv). At 30 dpv, both the *vtlR* mutant and M5-90Δ26 strain exhibited a significant reduction (~1 log_10_) in splenic bacterial loads compared to the PBS control (Figure 9A). Notably, the *vtlR* mutant conferred superior protection, with splenic bacterial counts significantly lower than those in the M5-90Δ26 group. At 45 dpv, the reduction in bacterial loads became more pronounced (1.5–2.0 log_10_ vs. PBS), with the *vtlR* mutant again outperforming M5-90Δ26 by an additional ~1 log_10_. Histopathological analysis of livers post-challenge further corroborated these findings (Figure 9B). At 30 dpv, PBS-control mice developed severe granulomas following virulence challenge, whereas no significant lesions were observed in the *vtlR* mutant group. M5-90Δ26-vaccinated mice exhibited only minor granulomas. By 45 dpv, granulomas persisted in PBS controls but were absent in both vaccinated groups after challenge. Collectively, these results demonstrate that the *vtlR* mutant elicits stronger and more durable immune protection than the conventional M5-90Δ26 vaccine.

## Discussion

It is well established that carbon sources are essential for bacterial growth and reproduction. In *Brucella*, the preferential utilization of carbon sources is closely associated with its pathogenicity. For instance, glucose uptake is critical for the intracellular survival of *B. abortus* and the establishment of chronic infection [20], while erythritol utilization serves as one of the key factors triggering abortion in pregnant livestock [22]. In this study, we found that the deletion of *vtlR* did not affect the growth of *B. melitensis* in nutrient-rich TSB medium but significantly impaired its ability to utilize L-fucose, D-glucose, and erythritol in PM, particularly L-fucose. In *B. abortus* strain 2308, Budnick and colleagues reported that three small proteins (BAB1_0914, BAB2_0512, and BAB2_0574) regulated by VtlR play a pivotal role in L-fucose utilization [14]. Through RNA-seq and RT-qPCR analysis, we identified that *B. melitensis* similarly regulates three homologous small proteins (RS04310, RS13565, and RS13280), suggesting that the L-fucose utilization defect in the *vtlR*  may result from their downregulated expression. However, the precise mechanism remains unclear. Notably, we observed that, in addition to L-fucose, the *vtlR* deletion significantly attenuated the utilization of glucose and erythritol in *B. melitensis*. This phenomenon implies that *vtlR* deficiency may disrupt common pathways of sugar metabolism or uptake mechanisms in *Brucella*. In *B. abortus* 2308, the presence of L-fucose upregulates genes related to sugar transport and metabolism [14], which might also influence the uptake and utilization of other carbohydrates. Furthermore, whether the impaired glucose and erythritol utilization in the *vtlR* mutant depends on the expression of these three small proteins was not thoroughly investigated in this study and warrants further exploration.

Our data demonstrate that VtlR plays a critical role in the intracellular survival of *Brucella* and its virulence in mice, which is consistent with previously reported findings in the *vtlR* mutant of *B. abortus* [13]. Based on the experimental data, we speculate that the mechanisms underlying VtlR-mediated intracellular survival and attenuated virulence of *Brucella* may involve the following aspects: (1) Impaired carbon source utilization in the *vtlR* mutant. *In vitro* experiments revealed that the *vtlR* mutant exhibits reduced capability to utilize glucose, erythritol, and fucose. Under cell culture conditions, particularly in DMEM medium where glucose serves as the primary carbon source, the intracellular survival defect of the *vtlR* mutant is likely attributable to its impaired carbon metabolism. In the mouse infection model, glucose transport and utilization are crucial for *Brucella* to establish chronic infection [20], suggesting that the attenuated virulence of the *vtlR* mutant in mice may similarly stem from its compromised carbon metabolic capacity. (2) The *vtlR* mutant shows diminished resistance to host-derived ROS and RNS. *In vitro* experimental data demonstrated that the *vtlR* mutant exhibited significant sensitivity to H_2_O_2_ and SNP—a finding not previously reported for *B. abortus*. Notably, Budnick et al. and colleagues found that VtlR-regulated target genes *BAB1_0914*, *BAB2_0512*, and *BAB2_0574* in *B. abortus* were significantly upregulated in the presence of H_2_O_2_ [14], suggesting an association between VtlR regulation and the oxidative stress response. Additionally, studies have revealed that LsrB, a homolog of VtlR in *Sinorhizobium meliloti* and *Agrobacterium tumefaciens* (which belong to the same order *Rhizobiales* as *Brucella*), is essential for bacterial adaptation to oxidative stress [23, 24]. Interestingly, VtlR from *B. abortus* could functionally complement the oxidative stress resistance defect in *A. tumefaciens lsrB/vtlR* deletion mutant [25], strongly supporting our finding that VtlR regulates oxidative stress resistance in *B. melitensis*. In subsequent experiments, we observed that infection of macrophages by the *vtlR* mutant significantly enhanced ROS release compared to the parental strain. This result further indicates that the impaired intracellular survival of the *vtlR* mutant may stem from oxidative stress-mediated killing. Similarly, during *Brucella* infection in animals, the host elevates oxidative stress levels—including the production of ROS and RNS—as a critical defense mechanism against bacterial infection [26]. Thus, we conclude that the attenuated oxidative stress resistance of the *vtlR* mutant is a key factor contributing to its reduced virulence.

As a transcriptional regulator, the attenuated virulence of *Brucella* caused by the deletion of *vtlR* primarily stems from its downstream target genes. Our experimental data reveal that in *B. melitensis*, VtlR predominantly downregulates the expression of four targets: three DUF1127 domain-containing proteins and the small RNA AbcR2. This finding aligns with previously reported VtlR-regulated targets in *B. abortus* [13]. However, plasmid-based complementation of these three DUF1127 proteins and AbcR2 in the *vtlR* mutant failed to restore its virulence (Figure 8). Consistently, literature reports indicate that neither individual nor combined deletion of these four genes affects *B. abortus* virulence [13]. Interestingly, we identified a fourth DUF1127 domain-containing protein in *Brucella*, encoded by the *RS13730* gene in *B. melitensis*, which exhibited significant upregulation in the *vtlR* mutant. In *B. abortus* 2308 strain, its homolog is encoded by *BAB2_0473*, but this gene was not detected in the microarray-based VtlR regulon [13], possibly due to differences in analytical sensitivity. Furthermore, comparative analysis of the *RS13730* promoter region with known VtlR-regulated targets revealed no presence of the reported VtlR-binding motif (CAATGCAGCCATGCA) [13]. Thus, it remains unclear whether *RS13730* is directly regulated by VtlR or compensatorily upregulated due to the downregulation of the other three DUF1127 proteins. In any case, both our study and previous research have identified VtlR as the primary regulator of DUF1127 domain-containing proteins. However, the DUF1127 proteins do not appear to be the key target gene through which VtlR influences *Brucella* virulence. Notably, we discovered that VtlR regulates several additional DEGs (Figure 7C), and whether these contribute to *Brucella* virulence warrants further investigation.

The significant attenuation of *Brucella* virulence caused by *vtlR* deletion has prompted our interest in developing the *vtlR* mutant as a potential vaccine candidate. Preliminary immunization studies demonstrated that the *vtlR* mutant confers superior protective immunity compared to the M5-90Δ26 vaccine strain. This enhanced protection may be attributed to the mutant's improved ability to activate macrophages, as evidenced by increased ROS production. While this study primarily focused on elucidating the role of VtlR in *Brucella* virulence, a comprehensive evaluation of the *vtlR* mutant as a vaccine candidate—including detailed analysis of humoral and cellular immune responses (particularly Th cell-mediated immunity) in inoculated animals—was not conducted. Notably, Th1-biased cellular immunity has been reported to play a critical role in protection against *Brucella* infection [27, 28]. Furthermore, whether the enhanced immunoprotection observed with the *vtlR* mutant stems from its superior ability to activate antigen presentation in macrophages and dendritic cells, as well as its residual virulence as an attenuated vaccine, remains to be investigated in following studies.

## Conclusion

In *B. abortus*, the transcription factor VtlR has been demonstrated to be essential for full virulence in a mouse infection model [13], but functional role of the VtlR in *B. melitensis* virulence remained undefined. Our experimental findings reveal that VtlR inactivation significantly impairs *B. melitensis* carbon metabolism, resistance to oxidative and nitrosative stress, intracellular survival, and virulence in mice. Furthermore, the Δ*vtlR* mutant strain demonstrated protective efficacy comparable to those conferred by the commercially available M5-90Δ26 reference vaccine. This study provides critical insights into the conserved regulatory role of VtlR in mediating virulence mechanisms across *Brucella* species.

## Supplementary Information

**Additional file 1. All primers used in this study**.

**Additional file 2. Sensitivity of the parental strain M5, the Δ*vtlR* mutant, and the complemented strain Δ*vtlR*-Rev to bactericidal agents**. **A** Polymyxin B susceptibility; **B** Sodium dodecyl sulfate (SDS) susceptibility; **C** Acid stress tolerance (low pH); **D** Sensitivity to natural goat serum. Statistical significance was assessed by one-way ANOVA (ns, not significant).

**Additional file 3. Adhesion and invasion capabilities of *Brucella* strains in host cells**. **A** HeLa cell infection model. **B** RAW 264.7 macrophage infection model. Statistical significance was assessed by one-way ANOVA (ns, not significant).

**Additional file 4. Verification of target gene overexpression constructs in the Δ*vtlR*** **mutant by PCR**.

## Data Availability

All data generated or analyzed during this study are included in this published article. The RNA-seq data have been deposited in the NCBI SRA database under BioProject accession number PRJNA1305112.
