# Supplementary figures and images for: The LysR-family transcriptional regulator VtlR coordinates carbon metabolism, oxidative and nitrosative stress resistance, and virulence in Brucella melitensis
Source: Vet Res. 2025 Nov 7;56:214. doi: 10.1186/s13567-025-01658-x (PMC12595884; doi:10.1186/s13567-025-01658-x)

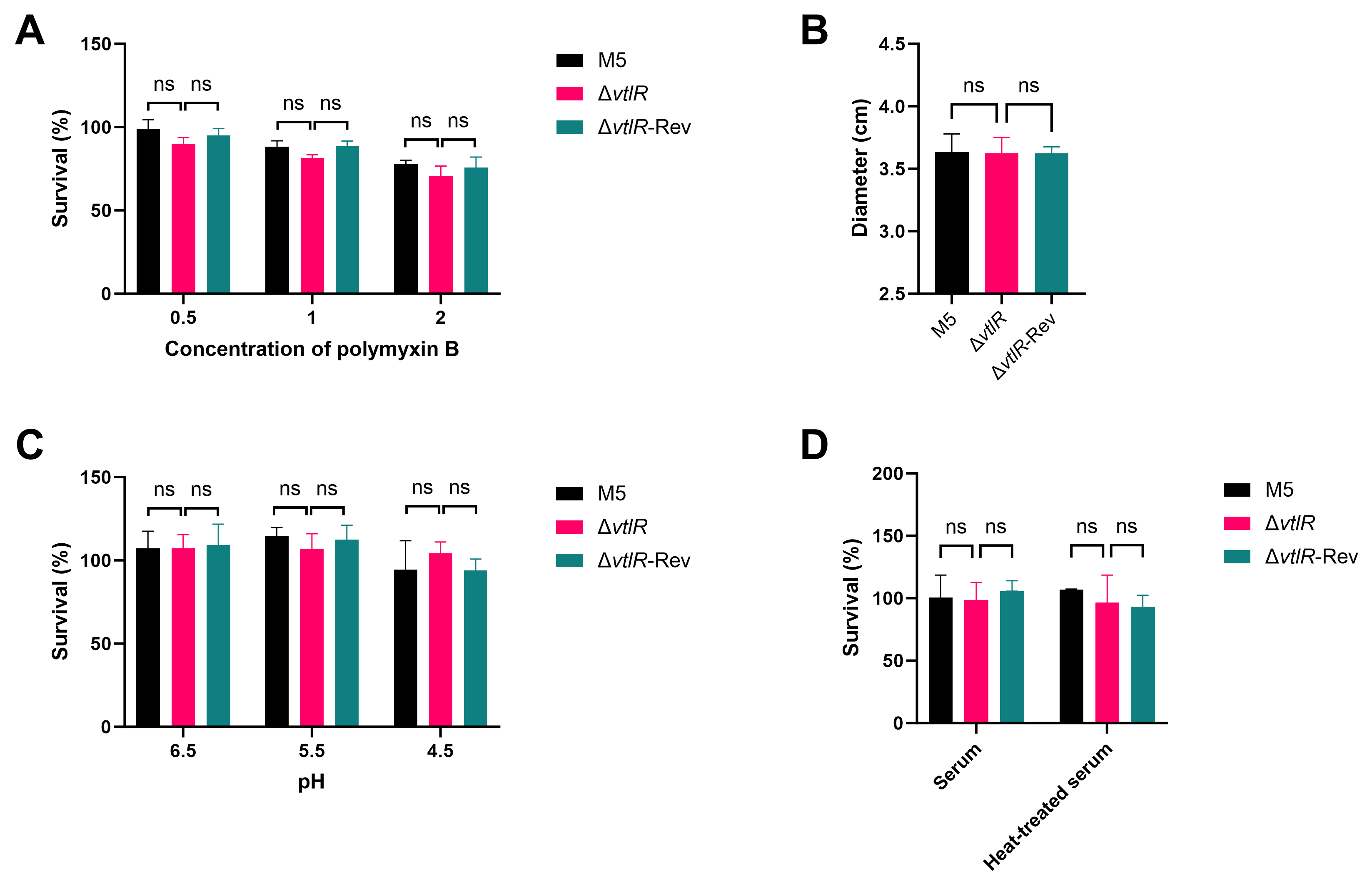

Supplement: Supplementary file 2 — Additional file 2. Sensitivity of the parental strain M5, the ΔvtlR mutant, and the complemented strain ΔvtlR-Rev to bactericidal agents. A Polymyxin B susceptibility; B Sodium dodecyl sulfate (SDS) susceptibility; C Acid stress tolerance (low pH); D Sensitivity to natural goat serum. Statistical significance was assessed by one-way ANOVA (ns, not significant). [file 13567_2025_1658_MOESM2_ESM.tif]

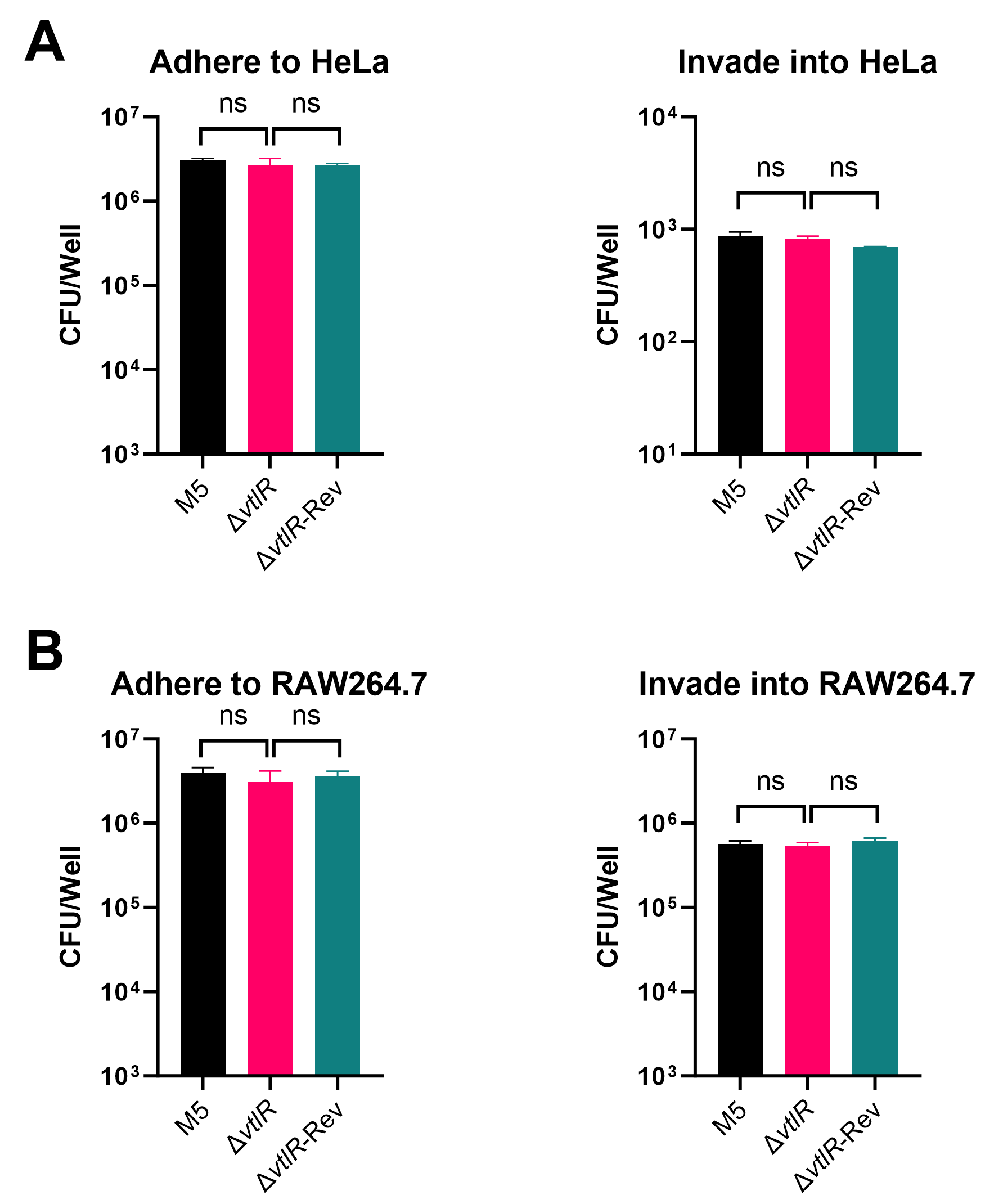

Supplement: Supplementary file 3 — Additional file 3. Adhesion and invasion capabilities of Brucella strains in host cells. A HeLa cell infection model. B RAW 264.7 macrophage infection model. Statistical significance was assessed by one-way ANOVA (ns, not significant). [file 13567_2025_1658_MOESM3_ESM.tif]

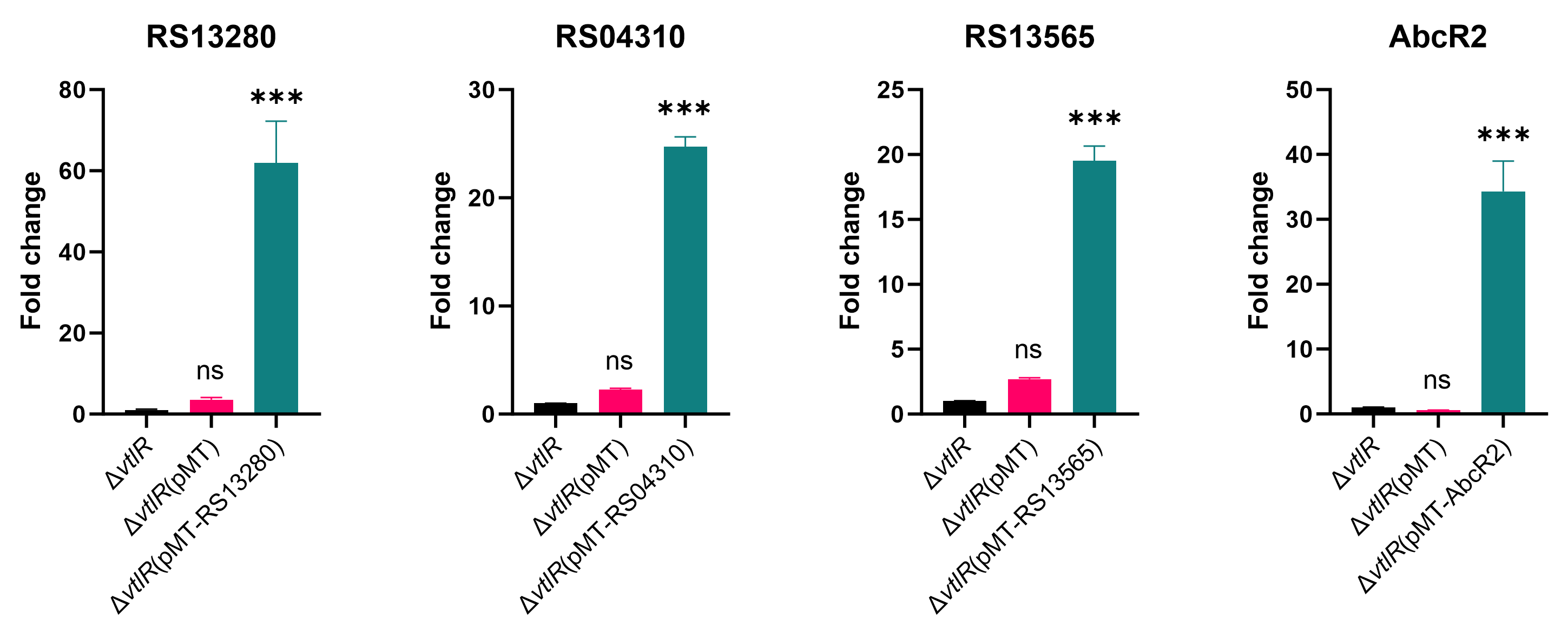

Supplement: Supplementary file 4 — Additional file 4. Verification of target gene overexpression constructs in the ΔvtlR mutant by PCR. [file 13567_2025_1658_MOESM4_ESM.tif]
